# Perspectives of Rectal Cancer Patients Undergoing Non-Operative Management (NOM): A Qualitative Study

**DOI:** 10.3390/curroncol33060348

**Published:** 2026-06-09

**Authors:** Armaghan Alam, Ameer Farooq, Farhad Udwadia, Manoj Raval, Ahmer Karimuddin, Terry Phang, Amandeep Ghuman, Carl Brown

**Affiliations:** 1 Division of General Surgery, Department of Surgery, University of Toronto, Toronto, ON M5T 1P5, Canada; army.alam@mail.utoronto.ca; 2 Division of General Surgery, Department of Surgery, Queen’s University, Kingston, ON K7L 2V7, Canada; 3 Division of Vascular Surgery, Department of Surgery, University of British Columbia, Vancouver, BC V6T 1Z4, Canada; 4 Division of General Surgery, Department of Surgery, St. Paul’s Hospital, University of British Columbia, Vancouver, BC V6Z 1Y6, Canada

**Keywords:** rectal cancer, non-operative management, patient perspectives, surveillance

## Abstract

There is growing interest in non-operative management (NOM) for rectal cancer patients who achieve a complete response to neoadjuvant therapy. The patients’ perspectives of these approaches are limited. Here, we describe a qualitative study where we conducted semi-structured interviews with fourteen rectal cancer patients, including seven men and seven women, who were successfully treated by NOM at our center between 2020 and 2022. The responses were analyzed using the constant comparative method. Four major thematic categories emerged: impact of rectal cancer diagnosis, treatment values, decision-making factors, and the impact of NOM surveillance. Avoidance of a stoma was a major theme in both determining patient treatment values as well as ultimately driving their decision-making. Trust in the treating physician was also found to be a major theme in decision-making. While the psychological burden of surveillance did emerge as a major theme, patients who did not have recurrence were still quite satisfied with their decision to pursue NOM. Limitations of this study include selection bias, the single-center design, and the lack of patients who ultimately experienced recurrence following NOM. As NOM of rectal cancer becomes more commonplace, understanding the patients’ perspectives will ensure appropriate counseling and shared decision-making.

## Simple Summary

Rectal cancer patients are traditionally treated with surgical resection of the rectum, referred to as total mesorectal excision, which comes with significant morbidity and mortality. In a subset of patients, non-operative management is being considered when there is a complete clinical response to neoadjuvant therapy. The perspectives of patients who select this non-operative approach are not well studied. We proposed a qualitative study to interview patients who had selected this approach to understand their decision-making and the psychological, physical, and emotional outcomes of their decisions. As non-operative management of rectal cancer becomes more commonplace, understanding the patients’ perspectives will ensure appropriate counseling and shared decision-making.

## 1. Introduction

Colorectal cancer (CRC) is the third most diagnosed cancer worldwide and the second most common cause of cancer mortality in men and women combined. Rectal cancer represents approximately 30–40% of all CRC cases [1,2]. Traditionally, patients with locally advanced rectal cancer have been treated with neoadjuvant therapy, followed by surgery with total mesorectal excision (TME) [3,4]. Radical resection is associated with significant morbidity and mortality, with long-term functional and psychosocial impacts [5].

Organ-preserving pathways have been developed for the treatment of rectal cancer in hopes of preserving good oncologic outcomes while mitigating the morbidity of radical resection. These include local excision as well as “watch and wait” or non-operative management (NOM) [6,7]. Currently, there are no well-established selection criteria that consistently identify patients for whom NOM will succeed [8,9]. Current protocols include rigorous surveillance with endoluminal evaluation and magnetic resonance imaging (MRI) every 3–6 months for 5 years [8].

While there has been significant interest in defining the clinical parameters that will allow for successful NOM, little work has been done to understand the patient experience through this process. Previous studies, such as those done by Gani et al. and Kennedy et al., have found that patients are willing to accept higher local recurrence rates and lower survival rates to allow for organ preservation [10,11]. However, these studies asked patients about their preferences in hypothetical scenarios rather than studying patients who were treated by NOM for rectal cancer. The frequent clinical/radiologic evaluations and the risk of local regrowth are potential sources of anxiety as well as time toxicity [12]. Accordingly, we designed a qualitative study to understand the perspectives of rectal cancer patients who had successfully undergone a NOM approach.

## 2. Materials and Methods

English-speaking participants over 18 years old who had undergone a NOM approach for rectal cancer were recruited using purposive sampling methods. There was no direct compensation or incentive offered to patients for contributing to this study.

To qualify for NOM, patients had a complete clinical response (cCR) with no palpable tumor on digital rectal exam (DRE), no residual tumor with a white–flat scar on endoscopy, no residual tumor on MRI, and no suspicious mesorectal or extra-mesorectal lymph nodes on MRI [11]. In addition, patients agreed to active surveillance, including digital rectal examination, endoscopy, and pelvic MRI at 3, 6, 9, 12, 18 and 24 months; CEA levels at 3, 6, 9, 12 15, 18, 21, and 24 months; and CT chest, abdomen, and pelvis at 6, 12, 18, and 24 months. Imaging is reviewed at our institution’s dedicated multidisciplinary conference with input from radiology, medical oncology, radiation oncology, and surgery. For the purposes of this study, only patients who had successful NOM after chemoradiation over a period of at least 6 months were included. Patients who required salvage surgery within these 6 months or who had local excision alone were excluded, as this may have constituted failure of neoadjuvant therapy. We were unable to recruit participants for this study who experienced recurrence outside of this initial exclusion period.

Approval for this study was granted by the University of British Columbia Research Ethics Board (H21-01034). Consent was obtained, and all interviews were audio-recorded and conducted over the video calling platform Zoom version 5.0 (Zoom Video Communications; San Jose, CA, USA). Interviews were conducted in a semi-structured fashion using a guide that had standardized questions with open-ended probe options that could be selected based on the flow of the interview. The interview guide was divided into 3 parts: (1) background and impact of rectal cancer on participants’ lives, (2) management and decision-making around choosing NOM, and (3) life after choosing NOM. The interview guide was developed in an iterative fashion. An initial list of questions was created to address the research goal of understanding the patients’ perspectives, choices, and outcomes with NOM. These were then reviewed with the authors of this paper and modified based on their expert clinical opinion. The questions on the finalized interview guide are shown in Table 1. Interviews were conducted by a medical student (author A.A.) who had significant prior experience in qualitative studies and semi-structured interviewing. He also had no relationship with or role in the treatment of the participants. Interviews began in July 2021, and recruitment concluded after thematic saturation was achieved. Recent analyses of thematic saturation have shown that 12 interviews are typically needed to reach higher degrees of saturation when using a threshold of ≤5% of new information being introduced in subsequent interviews [13]. For this study, thematic saturation was defined as 20% over the number of interviews after which no new themes emerged during the interview process, which was achieved at 14 interviews.

The interviews were then transcribed, made software-ready, and coded using NVivo version 12 (Lumivero (formerly QSR International); Denver, CO, USA). The coding guide was created based on a literature review and was revised after several rounds of iterative inductive and deductive analysis by two trained researchers (author AA and FU) until all themes were appropriately captured. The data analysis protocol described by Hrincu et al. was followed [14]. Transcripts were analyzed using Glaser’s constant comparative method for qualitative analysis to generate dominant themes and their constituent subthemes [15]. Authors A.A. and F.U. co-coded 15% of transcripts to ensure inter-rater reliability. Discrepancies in coding were reviewed until consensus was reached, and a Cohen kappa of >80% was calculated to ensure high intercoder reliability. This method utilized both peer debriefing and investigator triangulation to minimize the preconceptions of any one author. Primary themes in each thematic category were listed as the ones that were present in the greatest number of interviews, and secondary themes were the ones that followed. Themes were subsequently grouped into categories based on their inherent meanings. Illustrative quotes were included to highlight particularly salient themes.

## 3. Results

**Figure 1:**
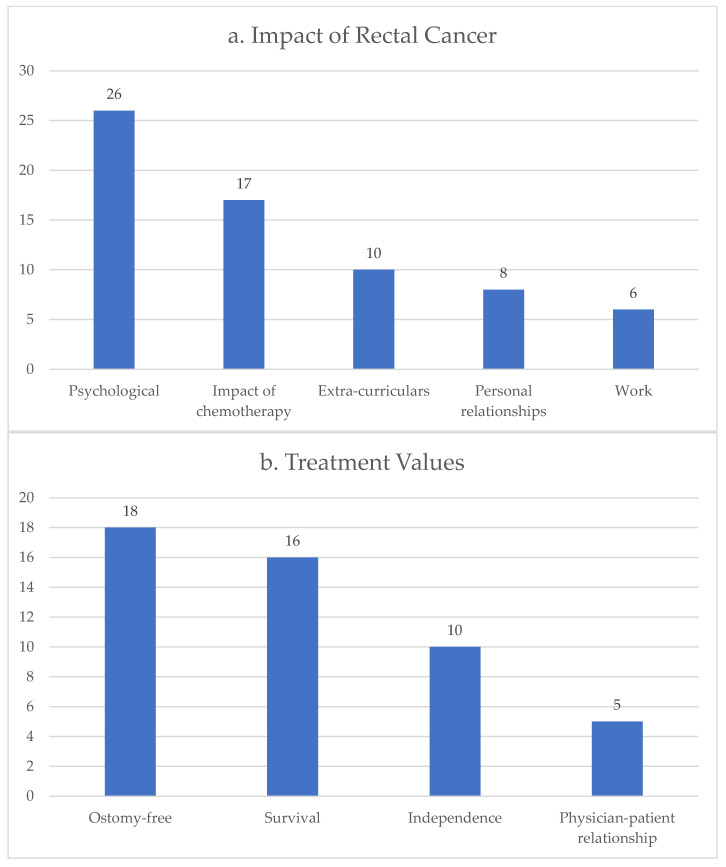

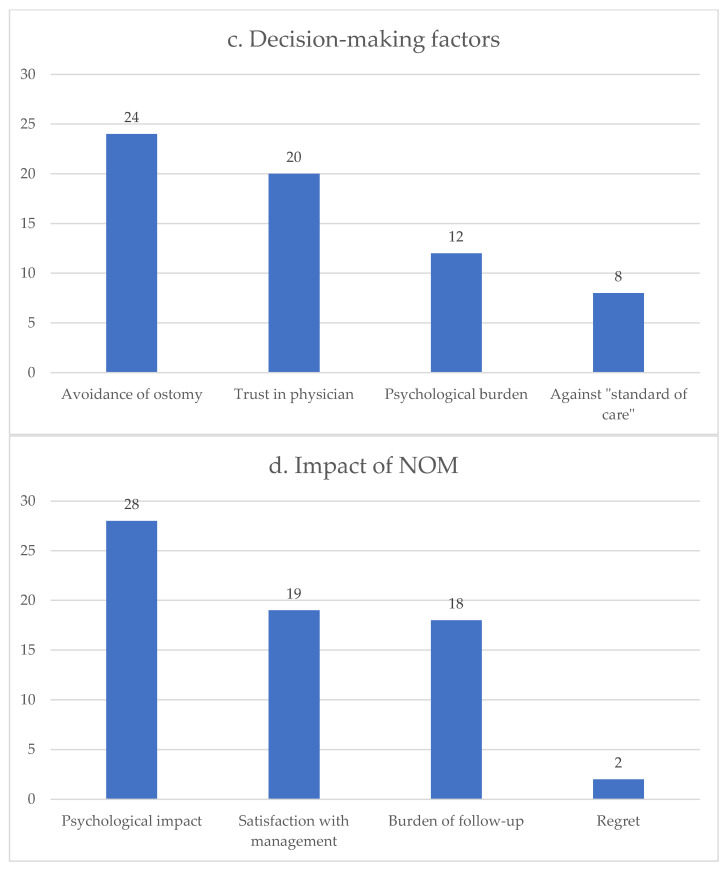
(**a**–**d**) Major categories of themes of importance to patients (frequency of respective themes).

**Figure 2:**
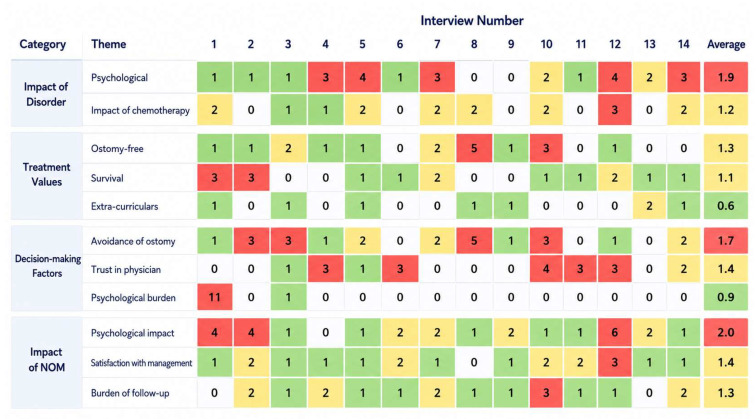
Heat map of themes important to NOM patients. Each number represents the frequency with which the theme was mentioned in the interview. Color coding was done only to help demonstrate this frequency: 0 is white, 1 is green, 2 is yellow, and 3 or more is red. The final column shows the average number of times that the theme was mentioned across all interviews, with 0 to 1 as green, 1.1 to 1.5 as yellow, and 1.6 to 2.0 as red.

Fourteen patients who met the inclusion criteria were recruited from across British Columbia. The cohort included seven males and seven females, with an age range between 27 and 88 years old. Included patients had treatment initiated between 2017 and 2021. Through the interview and coding process, we identified four major thematic categories of importance to patients based on frequency in the discourse: (1) impact of rectal cancer, (2) treatment values, (3) decision-making factors, and (4) impact of NOM (Figure 1a–d). A heat map of themes was developed based on the number of times each theme was mentioned per patient interview (Figure 2).

### 3.1. Impact of Rectal Cancer Diagnosis

The diagnosis of rectal cancer itself carried with it a significant psychological impact. This emerged as a primary theme, with 12 out of 14 participants expressing its importance. One participant noted:

“My favorite saying from Confucius is that every man has two lives, and the second one starts when he realizes he only has one life. And that was so big on me lying on that radiation table … it’s all about handling it mentally… it’s going to kill you way down the line, so yes, it’s a mental struggle like nothing I’ve ever had to overcome, but I did.”

The impact of chemoradiation emerged as a secondary theme in this category, with 9/14 participants discussing it as a significant aspect of their rectal cancer management. The impact was quite variable across participants. For some, chemoradiation was quite well tolerated:

“I live on a hill in… and I was walking hills in… until obviously the radiation burned me up to the point where I actually couldn’t, but that only happened for a week or so. I didn’t stop doing anything. I was doing it. I was really, really stubborn and really positive.”

For others, the chemoradiation had significant side effects, such as pelvic functional issues and pain.

“Well, it affected—during the treatment, it affected a lot—it affects your prostate, it affects your bladder. I mean, I tried to explain to somebody, it was like crapping razor blades. I mean, it gets pretty tender down there.”

### 3.2. Treatment Values

Patients were asked to define what was important to them in terms of their treatment values. In other words, what did patients hope that their treatment would achieve?

Under treatment values, both being ostomy-free and survival emerged as primary themes, with 10 of the 14 participants stating these as primary themes. With regards to being ostomy-free, one participant stated:

“That was the game changer for me, when he said you got to have a bag. Like, my friend had the same thing, colon cancer, and he didn’t have to have a bag. So that’s why I say, oh well, if I got to have surgery then I will. But as soon as he said your surgery will include a bag after that, it just killed it for me.”

Another participant noted succinctly:

“Really those two things were on my mind, I wasn’t thinking of anything else at all but staying alive and not having that ostomy.”

Patients valued the consequences of their decisions on their future survival and their relationships with their family.

“I just wanted to survive and enjoy the rest of my life. Even if I had to look a little different or even if it was going to hurt like hell, I was OK with that… I have two kids and I need to be around. I want to be there for their weddings and their graduations and all that kind of stuff. Maybe as a person, sometimes I don’t overanalyze those kinds of things that you mentioned, like the cosmetic side and that kind of thing [with regards to an ostomy].”

The secondary theme that emerged in the treatment value category was being able to continue with extra-curricular activities and work, expressed by 7/14 participants.

“I mean, it is—it does kind of put your life in a different mode of—you know, when you have to go have chemo and radiation—take chemo and radiation every day for, you know, two months, two and a half months. It does kind of set the scale for what you do.”

The diagnosis affected patients at various time points in their own life cycle:

“I think the interruption is like [during] a really exciting time of your life. The loss of control over how we start a family. Kind of the halt in my career and just developing in my profession in a meaningful way. This is the time where you’re looking at like purchasing a home, like starting a family. I was also diagnosed four days before I got married…the morning after my wedding I have six missed calls from medical providers.”

### 3.3. Decision-Making Factors

For decision-making factors with respect to NOM, avoidance of an ostomy was the primary theme (11/14 participants), with trust in the physician being secondary (8/14). With regards to avoiding an ostomy, one participant said:

“The opportunity for non-operative management was to feel safe and comfortable with the added bonus of I can continue to have my normal bowel function and aesthetically or visually look the same. Those things were nice to be able to preserve, at least while we can.”

Trust in the physician also emerged as a major factor in the decision-making process for a subset of patients (8 out of 14).

“When I’m thinking back on those eight years ago, Doctor… was incredibly over the top with that. I listened to him very closely. I trusted him, there was just something about his mannerisms that just made me trust him, and he trusted me. And we just kind of went down that path, you know?”

Several (5 out of 14 patients) underwent NOM after actively pursuing a second opinion or performing independent research with respect to their management:

“I’m part of this Facebook group called Colon Town… and that’s been a really great resource. They have a lot of—they have a whole like colorectal cancer university with lots of videos and resources there… And it seemed like a lot of the you know, a lot of the studies were saying that as long as you’re closely followed and if you require like that salvage surgery if the cancer comes back, the outcomes are you know, the same as if you had gone immediately in for surgery.”

Patients found it quite difficult to make the decision to pursue a NOM pathway. Patients struggled with the burden of having to decide in the face of uncertainty:

“Like, if it didn’t work, if the cancer came back, it wasn’t anyone else’s fault but mine because I didn’t follow the standard of care.”

Patients also struggled with balancing the statistical chances of successful NOM against the risk of recurrence:

“Because there’s certain statistics about like survival rates, you know, if you do the surgery then your survival rate is what? Ninety-two percent. And then like thirty percent of people, their cancer does come back. But then, for me, like statistically, I shouldn’t have even got this in the first place because some super high percentage of people are like over sixty-five when they get it. So, navigating statistics was very weird as an individual. Like, I can understand it maybe as a doctor or like a public health person. You have like data that’s in an aggregate. But when you’re just one person, it’s not, like you use it to base your decision but it’s only indicative because you’re only one person, right? So you don’t know which one … category you’re going to fall into.”

Patients were also confronted with considerable differences in provider opinion on management:

“He was livid. He was—I couldn’t believe it. I was sitting in a chair. I had my legs crossed. And I was looking at him and he was bouncing off the wall. And, “it’s going to come back, it’s going to kill you. I don’t know what the hell you guys are doing. I don’t know what the hell those doctors at St. Paul’s are doing. We got to have a meeting.” And I just like, I looked at him. I went, “wow.” And he stopped. He looked at me and I went, “you really got to settle down young man.”… That was the biggest pitfall was his, like, you guys are crazy. But Doctor… and I were going, I don’t think so.”

One of the patients also expressed concerns about how rectal cancer treatment might impact her fertility.

“That’s interesting in terms of my thoughts about non-operative management. I’d say from a fertility perspective early on it seemed to me that it was a very kind of clear-cut choice that the treatment pathway that would give me the best quality of life and best opportunity for bowel function preservation would be to have radiation followed by chemo and then if required, a surgery. … At that time when I heard that the chance of a complete response was 30%. I really dismissed that as a possibility; it just seemed far too wishful when I was even in that position at all. But knowing that radiation followed by chemo was the best course and knowing that the side-effect associated with that of permanence was that I wouldn’t be able to carry a pregnancy.“

### 3.4. Impact of NOM Pathway

For the final category, impact of NOM, the primary theme that emerged was the psychological burden associated with the pathway, expressed by 13/14 participants. One participant noted:

“Coming to Vancouver for my appointments is always a stressful time, because what if they find something on the scan? What if they find something in the MRI? What if there’s something in my blood work? What if? Is this going to be lingering in my head for the rest of my life? What are the chances of this reoccurring… it consumes people in a negative way… You know, it’s just what if this happened? What if that happened? But it didn’t, so I’m quite grateful for that. And I’m quite happy with the choice I made to select the non-operative path.”

The other primary theme in this category was the satisfaction participants felt with their management. Overall, 13 of 14 patients were satisfied with this treatment modality.

“Yeah. No, I, you know, every morning I wake up and I say, I’m healthy. I’ve never been this healthy. It is amazing. I’m very confident that more people will benefit from not getting the surgery if it can be avoided.”

Secondarily, 12 of 14 did discuss the burden of follow-up that was imposed by this pathway.

“In the first year I had re-checkup every three months. Every three months there’s blood, CT, MRI, and Doctor… went in the back with the mirrors, you know, and looked at the real picture of it. And every time it was OK we waited for the next three months. And then now in the last year it’s every six months now.”

## 4. Discussion

In this study, we investigated the perspectives of rectal cancer patients who successfully underwent a NOM pathway. To be a candidate for NOM, they were treated with either long-course chemoradiation or long/short-course radiotherapy combined with total neoadjuvant therapy. After treatment, patients who were found to have a cCR were offered a rigorous surveillance program instead of radical resection. Through our qualitative approach, four overarching themes emerged: the burden of the rectal cancer diagnosis itself, treatment values patients hold, the decision-making factors patients use when deciding treatment, and the impact of the NOM pathway itself. While survival was a major theme, avoidance of a stoma was the predominant theme that emerged both as an important treatment value as well as a decision-making factor. The psychological burden of the NOM pathway also emerged as an important issue for rectal cancer patients. Despite the psychological burden incurred by patients in a NOM pathway, the patients interviewed in whom NOM was successful at the time of their interview generally expressed satisfaction with their choice of treatment. Our manuscript provides important information for healthcare providers counselling patients eligible for a NOM pathway. Beyond the specifics of rectal cancer, our paper provides unique insights into the challenges for cancer patients embarking on a non-operative pathway.

While there is little narrative data on patient experience with organ preservation for rectal cancer, there are similar experiences in patients with prostate cancer. In a recent integrative review by Dickey et al., the majority of studies indicated that patients who received a watchful waiting approach reported low levels of anxiety and depression, as well as an overall decreased negative impact on quality of life, when compared to other treatment modalities [16]. However, this review also notes that, in two out of the five studies included, there were no significant differences in anxiety and depression. This was replicated in a study not included in the review by Steinech et al., which measured nine psychological variables in patients that were treated by radical prostatectomy versus watchful waiting, where watchful waiting was associated with similar outcomes in all of them [17]. Altogether, these variable results have further highlighted the importance of researching the psychological impacts of NOM.

That being said, a very recently published paper by Hilty Chu et al. does specifically explore the experience of rectal cancer patients who selected NOM [18]. In their study, they interviewed 15 patients at a single institution who were a median of 5 years out from their decision to pursue NOM, with a key difference that five patients had local regrowth, four had distal metastases, and one had a permanent stoma. Their study highlights that, while most patients were satisfied, a few had retrospective regret, addressing the experience of those who did have recurrence, which our study lacks. They do explain that these patients felt that no intervention or discussion would have altered their decision. Similar to our study, they also mention the significance of trust in their healthcare providers’ recommendations and the burden of surveillance/follow-up. Of note, it is unclear why in both studies, despite the burden and anxiety associated with surveillance, patients are generally satisfied with NOM. Further studies should explore this, but based on our results, we propose that, for these patients, the avoidance of surgery and an ostomy far outweighs the burden of active surveillance.

One vital difference when comparing the results of Hilty Chu et al.’s study and ours was that they commented on the fact that their patients overwhelmingly entered surveillance viewing surgery as an “inferior option”, and in some cases, “several patients did not recall discussions of surgery either before treatment or after achieving a cCR.” This was not a finding shared in our study, highlighting the significant importance of communication from healthcare providers regarding treatment options and alternatives, especially since, in both studies, trust in the healthcare provider in choosing NOM was a major decision-making factor. Strokes et al. explore the role of trust in the physician–patient encounter and highlight that, although fundamental to patient care, trust is declining [19]. They conclude in their scoping review that interventions aimed at improving shared decision-making can improve trust. In considering NOM, it is therefore crucial that patients are provided with unbiased and clear information regarding treatment options, enabling shared decision-making and improving trust in these settings.

From our study, NOM patients seem to experience two moments of significant psychological burden. The first is when NOM patients must initially make the decision to forgo radical surgery. Patients expressed the burden of making this decision and the potential consequences, real or imagined. In this regard, they struggled with fully understanding the competing risks and meaningfully integrating these into their decision-making. Patient decision-making is supported by ongoing research in decision aids and consent procedures. Various studies highlight the importance of more detailed and individualized information provided to patients, as well as acknowledging the wide disparity in patient preferences when it comes to treatment choice [20,21].

The second psychological burden occurred when patients presented for surveillance imaging or endoscopy. While patients did endorse the fear and anxiety that these assessments created, 13 of 14 patients expressed satisfaction with their ongoing care. Patients stated that there was anxiety about the possibility of recurrence, but this was balanced out by the reassurance and trust that patients reported in their surgeons, as well as the personal utility in avoiding surgery and its impact on their quality of life. We interpret this cautiously as none of the patients included in this study had a recurrence, and therefore, satisfaction rates in these patients may differ.

As NOM is increasingly sought by young patients, issues not typical in elderly patients arise. Of note, a recent study has found that there is no additional oncological risk in patients under the age of 50 years when following a watch-and-wait strategy after a cCR to chemoradiation, and therefore, NOM should be discussed with younger patients [22]. Specifically, in our study, only one young female patient discussed concerns about fertility, and although we cautiously interpret this exploratory finding, chemoradiation does have a significant impact on fertility. The American Society of Clinical Oncology (ASCO) has published guidelines surrounding fertility preservation for patients with cancer [23]. These include consideration of sperm, oocyte and embryo cryopreservation and potentially surgical procedures, such as oophoropexy [24]. As patients with rectal cancer that may be curable by surgery alone are counselled about neoadjuvant therapy and organ preservation, fertility must be part of the discussion in women and men considering having children.

Our findings support the concept of support around “survivorship”. Institutions should have strategies in place to help deal with both logistical and psychosocial aspects of active surveillance. At our institution, a nurse-navigator is a key point of contact for patients. The nurse-navigator keeps diligent track of upcoming tests and bloodwork. In addition, patients can reach out to our nurse-navigator with questions and concerns. Nurse-navigators have been found to improve processes of care and patient experience [25]. Institutions should also consider the creation of survivorship groups as well as survivorship packages, such as the ASCO survivorship care plan [26].

This study has several limitations. Firstly, our sample size is small and cannot be used to generalize perspectives across all NOM patients. Furthermore, all our participants came from a single center and, accordingly, may have been influenced by its culture and its healthcare providers’ communication style, limiting external transferability. The study by Hilty Chu et al. mentioned above, however, does strengthen the external validity and generalizability of the results, as we had similar findings at two different institutions in North America. Secondly, patients who have been deemed eligible for a NOM pathway are selected, in part, based on their commitment to comply with rigorous surveillance. In other words, patients included in this study are likely to be inherently different than patients who opted for radical resection, both psychosocially and with respect to tumor burden, leading to selection bias. Similarly, we did not collect information as to the reasoning behind why patients declined to participate in this study, which may impact sample representativeness. Thirdly, recall bias may impact the results, as some patients began treatment as early as 2017, making the retrospective interval considerable. Lastly, none of the patients in this study experienced recurrence, which may inflate satisfaction rates and lead to different perspectives on NOM. Although we acknowledge this throughout our study, it is again important for us to clarify that our study primarily focuses on understanding the experience of patients in whom NOM has been successful instead of comparing the experience of successful to unsuccessful NOM. We do discuss the findings of a similar study above that does look at the experience of those with recurrence [18].

An important aspect of the NOM pathway that we did not study was the impact on providers in helping guide patients through this decision. Despite almost twenty years of data on NOM, many providers do not feel that NOM is “ready for prime time” [27]. In a qualitative study performed by Covelli et al., physicians interviewed felt that NOM should not be routinely offered to patients who have experienced a cCR. Further work should investigate physician attitudes around NOM and how that interacts with patient decision-making.

Finally, it is important to note that this was not a comparative study looking at the quality of life for patients who had upfront radical resection versus those who underwent NOM. As noted by Vailati et al., one can only meaningfully compare the quality of life outcomes if TME and NOM were both possible alternatives for all patients [28]. Future trials, such as the STARTREC trial, should focus on comparing these various approaches and their impacts on quality of life [29].

## 5. Conclusions

In conclusion, NOM patients must juggle several competing elements when choosing to undergo NOM, including avoidance of an ostomy, anxiety around their diagnosis, and trust in their healthcare provider. While they do bear a psychological burden during active surveillance, NOM patients who have not experienced recurrence are quite satisfied with their choice to forgo radical resection. Further studies should continue to elicit the patient and healthcare provider experience in undergoing new rectal cancer treatments and specifically focus on how best to support these patients in making shared and informed decisions around their care.

## Figures and Tables

**Table 1:** Summary of interview questions.

Question	Probe
Part 1: Background and Impact of Rectal Cancer on Participants’ Lives	
What is your age?	
With what gender do you identify?	
What is it like having a diagnosis of rectal cancer?	How has it impacted your life, work, relationships?
Part 2: Management and Decision-making Around Choosing NOM	
What is most important to you in terms of what you want your treatment to achieve (i.e., treatment values)?	Can be no scar (cosmesis), being cancer free, survival, faster recovery time, symptomatic control, less doctor check-ups, minimal pain/discomfort, autonomy, long term prognosis, etc.
How has your rectal cancer been managed so far?	Did you undergo any surgical or other procedures, if so, which? What is involved in the management of your cancer currently? (i.e., how often do you have follow-up visits, scans, tests, procedures?)
Why did you choose to pursue your particular treatment plan?	What factors motivated you to pursue this particular plan? Did you have any hesitations? Who was involved in that selection process? How were the operative and non-operative options explained to you at the time? Was the novelty or experimental nature of this approach explained to you?
Part 3: Life After Choosing NOM	
How has this management modality impacted your life since?	How has it impacted your quality of life (work, relationships, or other aspects)? What is your physical well-being like? (i.e., bowel, bladder, sexual function)
Are you happy with this management pathway? Why or why not?	What aspects of it have brought you the most satisfaction? How has this approach met your expectations?
What has been the hardest part of this management approach?	Looking back is there anything else that you wished you had known?

## Data Availability

The datasets generated during and/or analyzed during the current study are available from the corresponding author on reasonable request.

## Abbreviations

The following abbreviations are used in this manuscript:

NOM	Non-operative management
CRC	Colorectal cancer
cCR	Complete clinical response
TME	Total mesorectal excision
MRI	Magnetic resonance imaging
DRE	Digital rectal exam
CEA	Carcinoembryonic antigen
CT	Computed tomography
ASCO	American Society of Clinical Oncology
